# Obstetrics, Gynecology, Pelvic and Abdominal Surgery

**Published:** 1898-01

**Authors:** William Warren Potter

**Affiliations:** Buffalo, N. Y., Member New York State Medical Examining and Licensing Board


### OBSTETRICS, GYNECOLOGY, PELVIC AND ABDOMINAL SURGERY.

CONDUCTED BY WILLIAM WARREN POTTER, M. D., Buffalo, N. Y.,
Member New York State Medical Examining and Licensing Board.

### PORRO OPERATION SUCCESSFUL TO MOTHER AND CHILD.

F. BLUME, Pittsburg, (*Jour. Am. Med. Assoc.;* also quoted in *Am. Jour. Surg. and Gynec.*,) gives an account of a successful Porro operation. The patient was 38 years old, mother of four children. On examination, June 18, 1895, the uterus was found to correspond in size and position to an eight months' pregnancy. The pelvic cavity was almost completely blocked with a dense ovarian tumor. The cervix uteri, high up and pushed to the right, could only with difficulty be reached by passing the finger between the tumor and the pubes. An attempt to push the tumor back was not successful. The question arose: Could the tumor be removed without interrupting pregnancy? If so, immediate ovariotomy would probably be the safest method of procedure. Ovariotomy, however, before the uterus was emptied of its contents was deemed impossible on account of the location of the tumor and its inaccessibility. Condition of the patient being good, it seemed to be proper to postpone interference in the interests of the child. Dr. Blume advised the Porro operation with removal of the growth and decided to operate a week before term. Operation was, therefore, performed July 12, 1895. Long incision. Uterus delivered from the abdominal cavity, the upper part of the incision closed to prevent escape of the intestines. A rubber ligature loosely applied around the cervix, to be tightened in case of hemorrhage. Uterus incised, membranes ruptured and the child delivered. The cord cut between forceps and the child handed to an assistant. Cervix constricted with the rubber ligature, transfixing pins applied, the placenta removed and the uterus amputated. The tumor, which arose from the left ovary and was bound down by adhesions, was enucleated, lifted out, ligatured and cut away. Peritoneum sewed around the stump below the rubber ligature. Abdominal incision closed and stump dressed with iodoform and iodoform gauze. The tumor, which was larger than a child's head, proved to be a multilocular cyst. The patient made a splendid recovery. The child, a fine boy weighing nine pounds, was put to her breast on the third day following the operation and lactation continued uninterruptedly. Both are now in excellent health. Upon examination, the cervix uteri is found very small, attached to the abdomen and giving no inconvenience. No ventral hernia.

———

ECTOPIC PREGNANCY.

H. W. Longyear (*Ann. of Gyn. and Ped.*; quoted also in *Med. Standard*) reports seven cases of ectopic pregnancy and recapitulates as follows:

*Diagnosis.*—(1) In examining pelvic affections of women of fruitful age, the possibility of ectopic pregnancy should be in the mind of the examiner. (2) A sudden attack, in such a woman, of sharp pain in the abdomen, attended with faintness, from which the woman does not soon recover, as from ordinary faintness, should direct attention to ectopic pregnancy as a possible cause.

(3) Distension of the bowels by flatus is characteristic of intraperitoneal hemorrhage. (4) Free fluid in the abdominal cavity can usually be demonstrated by percussion with change of position, in cases of intraperitoneal rupture. (5) The presence of intraperitoneal blood cannot be satisfactorily demonstrated by vaginal examination, as can the hematocele held tightly within the folds of the broad ligament, while in the pelvic and abdominal cavities the blood flows loosely and, except in rare instances, imparts no characteristic sensation to the examining fingers. (6) Pelvic hematocele, of traumatic origin, is usually, if not always, the result of a ruptured ectopic pregnancy. (7) A low febrile action, which is out of proportion to the other symptoms, is characteristic of ectopic pregnancy. (8) General peritonitis is not a result of ectopic pregnancy. (9) The discharge from the uterus, if a decidual membrane, is a valuable sign and when accompanied by unusually irregular menstruation, is of still more value.

*Treatment.*—(1) In all cases of intraperitoneal rupture, operate as soon as the diagnosis is made; do not wait for reaction to occur. Support such patients before and during the operation by application of heat to the extremities and back, transfusion of normal saline solution, rectal use of beef tea and saline solution and strychnia, nitroglycerine and digitalin by hypodermic injection. (2) If the hemorrhagic blood cannot be quickly removed, let it alone and use drainage. Do not flush the abdominal cavity to remove blood. The peritoneum will absorb the blood with much less danger than is caused by such manipulation. (3) Use silkworm gut "en masse" to close the abdominal wound, in case there has been great loss of blood, as the buried animal suture is not readily absorbed where such depletion has occurred. (4) Extraperitoneal hematocele is usually self-limiting and will almost always result in the death of the fetus and the recovery of the patient. (5) In cases that go on to full term, operate at the end of pregnancy to save the child. Operate through the abdomen, drain through vagina and leave the placenta to come away by disintegration. (6) Electricity should be used only in cases so situated as to preclude the possibility of securing proper surgical treatment.

---

### THE VALUE OF STERILISED MILK.

BARTON (*British Med. Journal;* also quoted in *Phila. Polyclinic*). The author, in a careful investigation of the subject, arrives at the following conclusions: (1) Completely sterilised milk, if administered without any fresh milk, will undoubtedly, sooner or later, produce scurvy. (2) Milk that is raised to the boiling point, or better, to within two degrees of the boiling point, and maintained there from for five to ten minutes, is comparatively sterilised and will never produce scurvy, and is almost free from pathogenic organisms. (3) Completely sterilised milk, if administered at once in perfectly clean bottles, spoons or cups can be relied upon as being free from any pathogenic microörganisms. (4) The heating of milk alters very little, if at all, its nourishing qualities. (5) All kinds of sterilised milk, if free from added chemicals, can become foul as quickly, if not more quickly, than ordinary fresh milk. (6) All sterilised milk that is put into hermetically sealed vessels, and which can keep fresh in them for many days, will produce scurvy unless some fresh food is administered daily. (7) Milk that is boiled directly over a fire will undoubtedly cause constipation. If the milk be placed in a vessel containing water, and the water be brought to the boiling point, its antiscorbutic properties are not lost and it does not cause constipation.

---

### THE OBSTETRICAL TREATMENT OF PUERPERAL ECLAMPSIA.

According to P. Drejer, (*St. Louis Clinique,*) the best treatment in puerperal eclampsia, both for mother and infant, is speedy delivery, whether labor has commenced or not. The best method of doing this is that which any medical man can employ, and for this and other reasons the author does not recommend Dührssen's plan (by incision), but prefers simple dilatation. Hegar's dilators are used in the first instance; then when the cervical canal will admit one finger the rest of the dilation is carried out manually and delivery is completed by the method of Braxton Hicks. The colpeurynter is not much used in Norway, for the marked variations in temperature are apt to interfere with caoutchouc dilators. Bimanual compression is applied to the uterus for about one hour after the removal of the placenta, and so hemorrhage is prevented. Drejer gives details of three cases in which this method was employed.

---

### INTEREST IN PLASTIC SURGERY.

Joseph Price (*Va. Med. Monthly*) says: "It is to be regretted that there is not a stronger interest in plastic surgery. If the profession would take the same interest, devote the equivalent in time, manifest the same enthusiasm in the practical study of plastic surgery they do to intraperitoneal disturbances, the result would be the relief of many sufferers, the saving of many mothers from permanent invalidism. No man can be a good abdominal and pelvic surgeon without a thorough knowledge of obstetrical science and without a bedside experience in the practice of its art; and few men give that study to practical obstetrics which enables them to do good plastic surgery.

"It is important, in fact absolutely essential, to be thoroughly familiar with the female pelvis and maternal soft parts before, during and after childbirth. There must be a careful study of the female perineum to enable the surgeon to restore to normal relations structures injured in childbirth. Throughout labor it is important to study the normal perineum; immediately after labor, by inspection and by digital examination, to determine the precise nature and extent of injuries, if any, resulting from the delivery. It is then you can determine all injuries of pelvic fascia and muscle, even those of the levator ani. You can also become informed of certain deep mutilations, due to the premature application of forceps. The injudicious and indiscriminate use of obstetrical forceps has for a long time been responsible for distressing and not easily repaired injuries of the pelvic floor. Those familiar with practical obstetrics rarely use obstetrical forceps—only in about 2 to 4 per cent., even in consulting work. As the obstetrician's experience broadens, as his work increases, his use for forceps diminishes, so does his infantile mortality. The schools are largely responsible for the too frequent use and abuse of forceps. The real character and extent of uncomfortable conditions, sequelæ of injuries to the pelvic floor, uterine decensus, posterior displacements, cysts and rectocele are scarcely recognised by many engaged in obstetrical practice.

"Primarily, I have but little patience with a certain large class of so-called operators who think they can do a perfect operation if they possess a pair of scissors and needle and a noisy needle-holder. I rejoice that all wrangling has ceased over one, two or three stitch methods of operation, so commonly practised a few years ago. The study of all the injuries should be prosecuted in a large dispensary service, where the injuries are studied carefully, repaired carefully and watched after the operations. A good number of such patients return to the dispensary for a physician to attend them in their approaching confinement. Here, again, he has an opportunity to study the repaired perineum, its tolerance to a perfect test of operations practised, also the nature of recurring tears."

### PRURITUS OF THE VULVA.

After as far as possible eliminating the cause, Dr. Morain (*N. Y. Med. Jour.*) orders vulvar lotions to be used night and morning of very hot water, to which 1 per cent. of chloral, coal tar or aromatic vinegar has been added. In addition to this, the affected region should be painted with the following solution :

| | | |
|---|---|---|
| ℞ | Cocain. hydroch. | gr. xv |
| | Aq. dest. | gr. 150 |

Dr. Morain also makes use of the following ointments :

| | | |
|---|---|---|
| ℞ | Menthol | gr. 45 |
| | Olive oil | gr. xv |
| | Lanolin | gr. 90 |
| ℞ | Potass bromide, | |
| | Salicylic acid | aa. gr. xv |
| | Glycerole of starch | gr. 300 |
| | Calomel | gr. vj |
| | Ext. belladon. | gr. iij |

He also recommends the following solution, which is to be used as a lotion :

| | | |
|---|---|---|
| ℞ | Mercury bichloride | gr. xxx |
| | Alcohol | gr. 150 |
| | Rose water | gr. 600 |
| | Aq. dest | ℥xiv |

If these remedies fail, says Dr. Morain, electricity, either the continued or the interrupted current, should be tried. In particularly rebellious cases, when the itching resists all kinds of treatment, resection of the tissues of the affected parts should be resorted to.

---

### ANTE-OPERATIVE EXAMINATION OF URINE.

Dr. L. S. McMurtry, Louisville, (*Am. Jour. Surg. and Gynec.*,) says that a systematic examination of the urine of all women who come to the surgeon for operation will show pus in the urine in a very large proportion of cases. This should not deceive the operator —it may be due to leucorrhea, to gonorrhea and the like, or be purely transient, disappearing as soon as the operation has been performed. Unless there be quite a large amount of pus, it is, therefore, not necessary to spend useless time and energy in seeking a remote cause, provided a possible source of the discharge is present.

